# Crop Classification by Forward Neural Network with Adaptive Chaotic Particle Swarm Optimization

**DOI:** 10.3390/s110504721

**Published:** 2011-05-02

**Authors:** Yudong Zhang, Lenan Wu

**Affiliations:** School of Information Science and Engineering, Southeast University, Nanjing 210096, China; E-Mail: zhangyudongnuaa@gmail.com

**Keywords:** artificial neural network, synthetic aperture radar, principle component analysis, particle swarm optimization

## Abstract

This paper proposes a hybrid crop classifier for polarimetric synthetic aperture radar (SAR) images. The feature sets consisted of span image, the H/A/α decomposition, and the gray-level co-occurrence matrix (GLCM) based texture features. Then, the features were reduced by principle component analysis (PCA). Finally, a two-hidden-layer forward neural network (NN) was constructed and trained by adaptive chaotic particle swarm optimization (ACPSO). *K*-fold cross validation was employed to enhance generation. The experimental results on Flevoland sites demonstrate the superiority of ACPSO to back-propagation (BP), adaptive BP (ABP), momentum BP (MBP), Particle Swarm Optimization (PSO), and Resilient back-propagation (RPROP) methods. Moreover, the computation time for each pixel is only 1.08 × 10^−7^ s.

## 1. Introduction

The classification of different objects, as well as different terrain characteristics, with single channel monopolarisation SAR images can carry a significant amount of error, even when operating after multilooking [1]. One of the most challenging applications of polarimetry in remote sensing is landcover classification using fully polarimetric SAR (PolSAR) images [2].

The Wishart maximum likelihood (WML) method has often been used for PolSAR classification [3]. However, it does not take explicitly into consideration the phase information contained within polarimetric data, which plays a direct role in the characterization of a broad range of scattering processes. Furthermore, the covariance or coherency matrices are determined after spatial averaging and therefore can only describe stochastic scattering processes while certain objects, such as man-made objects, are better characterized at pixel-level [4].

To overcome above shortcomings, polarimetric decompositions were introduced with an aim at establishing a correspondence between the physical characteristics of the considered areas and the observed scattering mechanisms. The most effective method is the Cloude decomposition, also known as H/A/α method [5]. Recently, texture information has been extracted, and used as a parameter to enhance the classification results. The gray-level co-occurrence matrices (GLCM) were already successfully applied to classification problems [6]. We choose the combination of H/A/α and GLCM as the parameter set of our study.

In order to reduce the feature vector dimensions obtained by H/A/α and GLCM, and to increase the discriminative power, the principal component analysis (PCA) method was employed. PCA is appealing since it effectively reduces the dimensionality of the feature and therefore reduces the computational cost.

The next problem is how to choose the best classifier. In the past years, standard multi-layered feed-forward neural networks (FNN) have been applied for SAR image classification [7]. FNNs are effective classifiers since they do not involve complex models and equations as compared to traditional regression analysis. In addition, they can easily adapt to new data through a re-training process.

However, NNs suffer from converging too slowly and being easily trapped into local extrema if a back propagation (BP) algorithm is used for training [8]. Genetic algorithm (GA) [9] has shown promising results in searching optimal weights of NN. Besides GA, Tabu search (TS) [10], Particle Swarm Optimization (PSO) [11], and Bacterial Chemotaxis Optimization (BCO) [12] have also been reported. However, GA, TS, and BCO have expensive computational demands, while PSO is well-known for its lower computation cost, and the most attractive feature of PSO is that it requires less computational bookkeeping and a few lines of implementation codes.

In order to improve the performance of PSO, an adaptive chaotic PSO (ACPSO) method was proposed. In order to prevent overfitting, cross-validation was employed, which is a technique for assessing how the results of a statistical analysis will generalize to an independent data set and is mainly used to estimate how accurately a predictive model will perform in practice [13]. One round of cross-validation involves partitioning a sample of data into complementary subsets, performing the analysis on one subset (called the training set), and validating the analysis on the other subset (called the validation set) [14]. To reduce variability, multiple rounds of cross-validation are performed using different partitions, and the validation results are averaged over the rounds [15].

The structure of this paper is as follows: In the next Section 2 the concept of Pauli decomposition was introduced. Section 3 presents the span image, the H/A/α decomposition, the feature derived from GLCM, and the principle component analysis for feature reduction. Section 4 introduces the forward neural network, proposed the ACPSO for training, and discussed the importance of using *k*-fold cross validation. Section 5 uses the NASA/JPL AIRSAR image of Flevoland site to show our proposed ACPSO outperforms traditional BP, adaptive BP, BP with momentum, PSO, and RPROP algorithms. Final Section 6 is devoted to conclusion.

## 2. Pauli Decomposition

### 2.1. Basic Introduction

The features are derived from the multilook coherence matrix of the PolSAR data [5]. Suppose:

(1) $$S\;=\;\left[\begin{array}{cc} S_{hh} & S_{hv}\\ S_{vh} & S_{vv} \end{array}\right]\;=\;\left[\begin{array}{cc} S_{hh} & S_{hv}\\ S_{hv} & S_{vv} \end{array}\right]$$

stands for the measured scattering matrix. Here *S_qp_* represents the scattering coefficients of the targets, *p* the polarization of the incident field, *q* the polarization of the scattered field. *S_hv_* equals to *S_vh_* since reciprocity applies in a monostatic system configuration.

The Pauli decomposition expresses the scattering matrix *S* in the so-called Pauli basis, which is given by the following three 2 × 2 matrices:

(2) $$S_{a}\;=\;\frac{1}{\sqrt{2}}\left[\begin{array}{cc} 1 & 0\\ 0 & 1 \end{array}\right],\;S_{b}\;=\;\frac{1}{\sqrt{2}}\left[\begin{array}{cc} 1 & 0\\ 0 & -1 \end{array}\right],\;S_{c}\;=\;\frac{1}{\sqrt{2}}\left[\begin{array}{cc} 0 & 1\\ 1 & 0 \end{array}\right]$$

Thus, *S* can be expressed as:

(3) $$S\;=\;aS_{a}\;+\;bS_{b}\;+\;cS_{c}$$

where:

(4) $$a\;=\;\frac{S_{hh}\;+\;S_{vv}}{\sqrt{2}},\;b\;=\;\frac{S_{hh}\;-\;S_{vv}}{\sqrt{2}},\;c\;=\;\sqrt{2}S_{hv}$$

An RGB image could be formed with the intensities |*a*|^2^, |*b*|^2^, |*c*|^2^. The meanings of *S_a_*, *S_b_*, and *S_c_* are listed in Table 1.

### 2.2. Coherence Matrix

The coherence matrix is obtained as [16]:

(5) $$T\;=\;[a,\;b,\;c]{\left[a,\;b,\;c\right]}^{T}\;=\;\left[\begin{array}{ccc} T_{11} & T_{12} & T_{13}\\ T_{12}^{*} & T_{22} & T_{23}\\ T_{13}^{*} & T_{23}^{*} & T_{33} \end{array}\right]$$

The average of multiple single-look coherence matrices is the multi-look coherence matrix. (*T*_11_, *T*_22_, *T*_33_) usually are regarded as the channels of the PolSAR images.

## 3. Feature Extraction and Reduction

The proposed features can be divided into three types, which are explained below.

### 3.1. Span

The span or total scattered power is given by:

(6) $$M\;=\;{\left|S_{hh}\right|}^{2}\;+\;{\left|S_{vv}\right|}^{2}\;+\;2{\left|S_{hv}\right|}^{2}$$

which indicates the power received by a fully polarimetric system.

### 3.2. H/A/Alpha Decomposition

H/A/α decomposition is designed to identify in an unsupervised way polarimetric scattering mechanisms in the *H*-α plane [5]. The method extends the two assumptions of traditional ways [17]: (1) azimuthally symmetric targets; (2) equal minor eigenvalues *λ*_2_ and *λ*_3_. *T* can be rewritten as:

(7) $$T\;=\;U_{3}\;\left[\begin{array}{ccc} \lambda_{1} & 0 & 0\\ 0 & \lambda_{2} & 0\\ 0 & 0 & \lambda_{3} \end{array}\right]\;U_{3}^{H}$$

where:

(8) $$U_{3}\;=\;\left[\begin{array}{ccc} \text{cos}\;\alpha_{1} & \text{cos}\;\alpha_{2} & \text{cos}\;\alpha_{3}\\ \text{sin}\;\alpha_{1}\;\text{cos}\;\beta_{1}\;\text{exp}(i\delta_{1}) & \text{sin}\;\alpha_{2}\;\text{cos}\;\beta_{2}\;\text{exp}(i\delta_{2}) & \text{sin}\;\alpha_{3}\;\text{cos}\;\beta_{3}\;\text{exp}(i\delta_{3})\\ \text{sin}\;\alpha_{1}\;\text{sin}\;\beta_{1}\;\text{exp}(i\gamma_{1}) & \text{sin}\;\alpha_{2}\;\text{sin}\;\beta_{2}\;\text{exp}(i\gamma_{2}) & \text{sin}\;\alpha_{3}\;\text{sin}\;\beta_{3}\;\text{exp}(i\gamma_{3}) \end{array}\right]$$

Then, the pseudo-probabilities of the *T* matrix expansion elements are defined as:

(9) $$P_{i}\;=\;\frac{\lambda }{\sum_{j=1}^{3}\lambda_{j}}$$

The entropy [18] indicates the degree of statistical disorder of the scattering phenomenon. It can be defined as:

(10) $$H\;=\;\sum_{i=1}^{3}-P_{i}\;{\text{log}}_{3}\;P_{i}\;0\;\leq \;H\;\leq \;1$$

For high entropy values, a complementary parameter (anisotropy) [1] is necessary to fully characterize the set of probabilities. The anisotropy is defined as the relative importance of the second scattering mechanisms [19]:

(11) $$A\;=\;\frac{P_{2}\;-\;P_{3}}{P_{2}\;+\;P_{3}}\;0\;\leq \;A\;\leq \;1$$

The four estimates of the angles are easily evaluated as:

(12) $$[\bar{\alpha },\;\bar{\beta },\;\bar{\delta },\;\bar{\gamma }]\;=\;\sum_{i=1}^{3}P_{i}[\alpha ,\;\beta ,\;\delta ,\;\gamma ]$$

Thus, vectors from coherence matrix can be represented as (*H*, *A*, *ᾱ*, *β̄*, *δ̄*, *γ̄*).

### 3.3. Texture Features

Gray level co-occurrence matrix (GLCM) is a text descriptor which takes into account the specific position of a pixel relative to another. The GLCM is a matrix whose elements correspond to the relative frequency of occurrence of pairs of gray level values of pixels separated by a certain distance in a given direction [20]. Formally, the elements of a GLCM *G*(*i*,*j*) for a displacement vector (*a*,*b*) is defined as:

(13) G (i, j) = |{(x, y), (t, v) : I(r, s) = i & I(t, v) = j}|

where (*t*,*v*) = (*x* + *a*, *y* + *b*), and |•| denotes the cardinality of a set. The displacement vector (*a*,*b*) can be rewritten as (*d*, *θ*) in polar coordinates.

GLCMs are suggested to be calculated from four displacement vectors with *d* = 1 and *θ* = 0°, 45°, 90°, and 135° respectively. In this study, the (*a*, *b*) are chosen as (0,1), (−1,1), (−1,0), and (−1,–1) respectively, and the corresponding GLCMs are averaged. The four features are extracted from normalized GLCMs, and their sum equals to 1. Suppose the normalized GLCM value at (*i*,*j*) is *p*(*i*,*j*), and their detailed definition are listed in Table 2.

### 3.4. Total Features

The texture features consist of 4 GLCM-based features, which should be multiplied by 3 since there are three channels (*T*_11_, *T*_22_, *T*_33_). In addition, there are one span feature, and six *H*/α parameters. In all, the number of total features is 1 + 6 + 4 × 3 = 19.

### 3.5. Principal Component Analysis

PCA is an efficient tool to reduce the dimension of a data set consisting of a large number of interrelated variables while retaining most of the variations. It is achieved by transforming the data set to a new set of ordered variables according to their variances or importance. This technique has three effects: It orthogonalizes the components of the input vectors so that uncorrelated with each other, it orders the resulting orthogonal components so that those with the largest variation come first, and eliminates those components contributing the least to the variation in the data set [21].

More specifically, for a given n-dimensional matrix *n × m*, where *n* and *m* are the number of variables and the number of temporal observations, respectively, the *p* principal axes (*p ≪ n*) are orthogonal axes, onto which the retained variance is maximal in the projected space. The PCA describes the space of the original data projecting onto the space in a base of eigenvectors. The corresponding eigenvalues account for the energy of the process in the eigenvector directions. It is assumed that most of the information in the observation vectors is contained in the subspace spanned by the first *p* principal components. Considering data projection restricted to *p* eigenvectors with the highest eigenvalues, an effective reduction in the input space dimensionality of the original data can be achieved with minimal information loss. Reducing the dimensionality of the *n* dimensional input space by projecting the input data onto the eigenvectors corresponding to the first *p* eigenvalues is an important step that facilitates subsequent neural network analysis [22].

**Figure 1.**
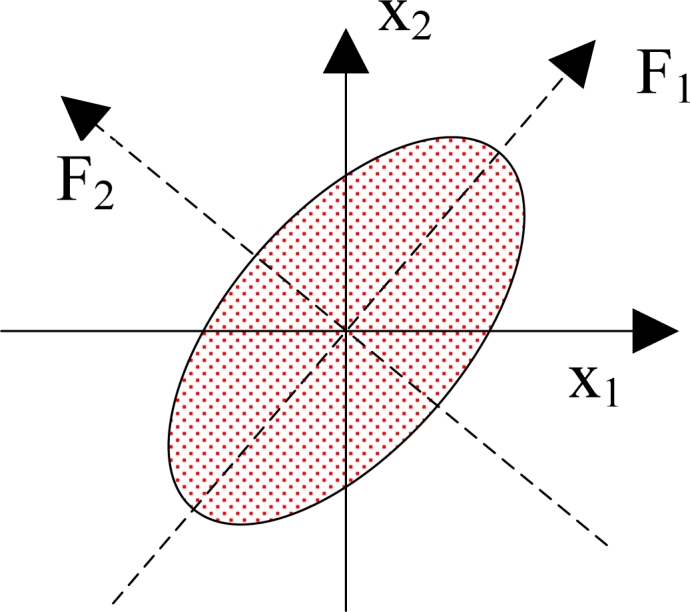
Geometric Illustration of PCA.

The detailed steps of PCA are as follows: (1) organize the dataset; (2) calculate the mean along each dimension; (3) calculate the deviation; (4) find the covariance matrix; (5) find the eigenvectors and eigenvalues of the covariance matrix; (6) sort the eigenvectors and eigenvalues; (7) compute the cumulative energy content for each eigenvector; (8) select a subset of the eigenvectors as the new basis vectors; (9) convert the source data to z-scores; (10) project the z-scores of the data onto the new basis. Figure 1 shows a geometric illustration of PCA. Here the original basis is {*x*_1_, *x*_2_}, and the new basis is {*F*_1_, *F*_2_}. After the data was projecting onto the new basis, we can find that the data focused along the first dimension of the new basis.

## 4. Forward Neural Network

Neural networks are widely used in pattern classification since they do not need any information about the probability distribution and the *a priori* probabilities of different classes. A two-hidden-layer backpropagation neural network is adopted with sigmoid neurons in the hidden layers and linear neuron in the output layer via the information entropy method [23].

**Figure 2.**
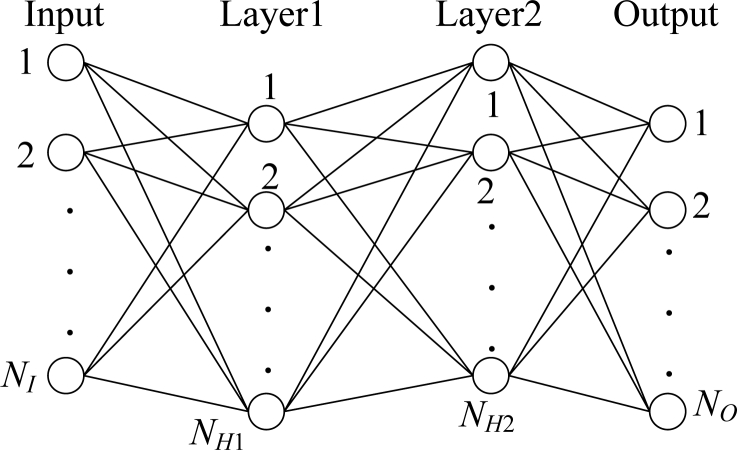
A three-layer neural network.

The training vectors are formed from the selected areas and normalized and presented to the NN which is trained in batch mode. The network configuration is *N_I_* × *N_H_*_1_ × *N_H_*_2_ × *N_O_*, *i.e.*, a three-layer network with *N_I_* neurons in the input layer, *N_H_*_1_ neurons in the first hidden layer, *N_H_*_2_ neurons in the second hidden layer, and *N_O_* neuron in the output layer (Figure 2). Their values vary with the remote-sensing area, and will be determined in the Experimental section.

### 4.1. Introduction of PSO

**Figure 3.**
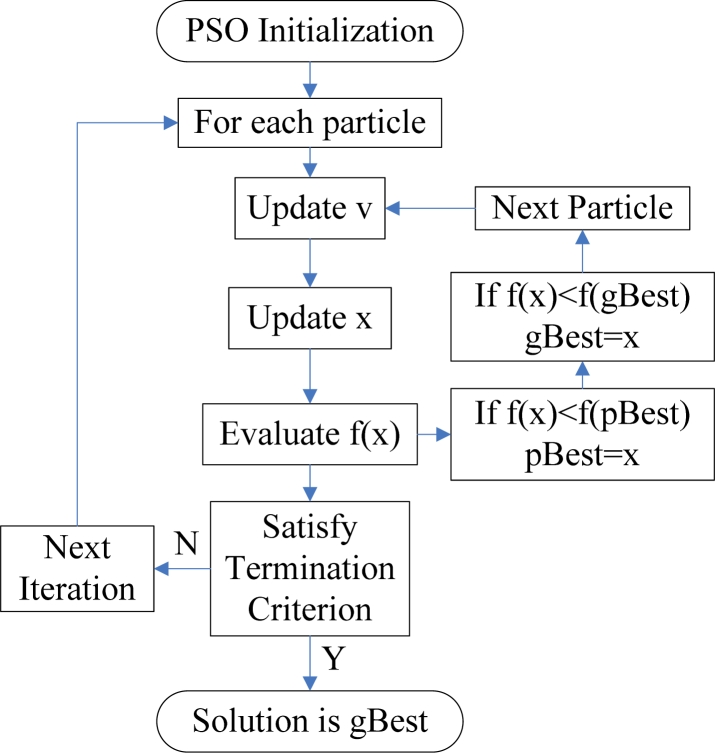
Flow chart of the PSO algorithm.

The traditional NN training method can easily be trapped into the local minima, and the training procedures take a long time [24]. In this study, PSO is chosen to find the optimal parameters of the neural network. PSO is a population based stochastic optimization technique, which is based on simulating the social behavior of swarm of bird flocking, bees, and fish schooling. By randomly initializing the algorithm with candidate solutions, the PSO successfully leads to a global optimum [25]. This is achieved by an iterative procedure based on the processes of movement and intelligence in an evolutionary system. Figure 3 shows the flow chart of a PSO algorithm.

In PSO, each potential solution is represented as a particle. Two properties (position *x* and velocity *v*) are associated with each particle. Suppose *x* and *v* of the ith particle are given as [26]:

(14) $$x\;=\;(x_{i1},\;x_{i2},\;\cdots ,\;x_{iN})$$

(15) $$v\;=\;(v_{i1},\;v_{i2},\;\cdots ,\;v_{iN})$$

where *N* stands for the dimensions of the problem. In each iteration, a fitness function is evaluated for all the particles in the swarm. The velocity of each particle is updated by keeping track of two best positions. One is the best position a particle has traversed so far. It is called “*pBest*”. The other is the best position that any neighbor of a particle has traversed so far. It is a neighborhood best and is called “*nBest*”. When a particle takes the whole population as its neighborhood, the neighborhood best becomes the global best and is accordingly called “*gBest*”. Hence, a particle’s velocity and position are updated as follows:

(16) $$v\;=\;\omega \cdot v\;+\;c_{1}r_{1}\;(p\text{Best}\;-\;x)\;+\;c_{2}r_{2}\;(n\text{Best}\;-\;x)$$

(17) x = x + vΔt

where *ω* is called the “*inertia weight*” that controls the impact of the previous velocity of the particle on its current one. *c*_1_ and *c*_2_ are positive constants, called “*acceleration coefficients*”. *r*_1_ and *r*_2_ are random numbers that are uniformly distributed in the interval [0,1]. These random numbers are updated every time when they occur. Δ*t* stands for the given time-step and usually equals to 1.

The population of particles is then moved according to Equations (16) and (17), and tends to cluster together from different directions. However, a maximum velocity *v*_max_, should not be exceeded by any particle to keep the search within a meaningful solution space. The PSO algorithm runs through these processes iteratively until the termination criterion is satisfied.

Let *NP* denotes the number of particles, each having a position *x_i_* and a velocity *v_i_*. Let *p_i_* be the best known position of particle *i* and *g* be the best known position of the entire swarm. A basic PSO algorithm can be described as follows:

Step 1 Initialize every particle’s position with a uniformly distributed random vector;
Step 2 Initialize every particle’s best known position to its initial position, *viz.*, *p_i_* = *x_i_*;
Step 3 If *f*(*p_i_*) < *f*(*g*), then update the swarm’s best known position, *g* = *p_i_*;
Step 4 Repeat until certain termination criteria was met
  Step 4.1 Pick random numbers *r*_1_ & *r*_2_;
  Step 4.2 Update every particle’s velocity according to formula (16);
  Step 4.3 Update every particle’s position according to formula (17);
  Step 4.4 If *f*(*x_i_*) < *f*(*p_i_*), then update the particle’s best known position, *p_i_* = *x_i_*. If *f*(*p_i_*) < *f*(*g*), then update the swarm’s best known position, *g* = *p_i_*.
  Step 5 Output *g* which holds the best found solution.

### 4.2. ACPSO

In order to enhance the performance of canonical PSO, two improvements are proposed as follows. The inertia weight *ω* in Equation (16) affects the performance of the algorithm. A larger inertia weight pressures towards global exploration, while a smaller one pressures towards fine-tuning of current search area [27]. Thus, proper control of *ω* is important to find the optimum solution accurately. To deal with this shortcoming, an “*adaptive inertia weight factor*” (AIWF) was employed as follow:

(18) $$\omega \;=\;\omega_{\text{max}}\;-\;(\omega_{\text{max}}\;-\;\omega_{\text{min}})/k_{\text{max}}\;\times \;k$$

Here, *ω*_max_ denotes the maximum inertial weight, *ω*_min_ denotes the minimum inertial weight, *k*_max_ denotes the epoch when the inertial weight reaches the final minimum, and *k* denotes current epoch.

The parameters (*r*_1_, *r*_2_) were generated by pseudo-random number generators (RNG) in classical PSO. The RNG cannot ensure the optimization’s ergodicity in solution space because they are pseudo-random; therefore, we employed the Rossler chaotic operator [28] to generate parameters (*r*_1_, *r*_2_). The Rossler equations are as follows:

(19) $$\{\begin{array}{c} \frac{dx}{dt}\;=\;-(y\;+\;z)\\ \frac{dy}{dt}\;=\;x\;+\;ay\\ \frac{dz}{dt}\;=\;b\;+\;xz\;-\;cz \end{array}$$

**Figure 4.**
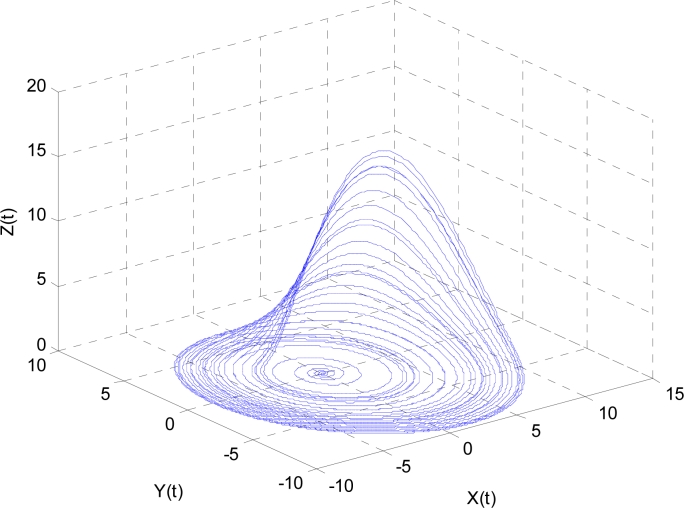
A Rossler chaotic number generator with *a* = 0.2, *b* = 0.4, *c* = 5.7.

Here *a*, *b*, and *c* are parameters. In this study, we chose *a* = 0.2, *b* = 0.4, and *c* = 5.7. The results are shown in Figure 4, where the line in the 3D space exhibits a strong chaotic property called “spiral chaos”.

**Figure 5.**
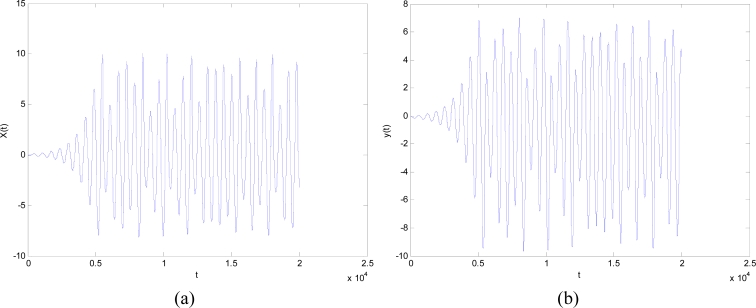
Chaotic sequence of (**a**) *x*(*t*) and (**b**) *y*(*t*).

The dynamic properties of *x*(*t*) and *y*(*t*) are shown in Figure 5, where *x*(*t*) and *y*(*t*) satisfy both ergodicity and randomness. Therefore, we let *r*_1_ = *x*(*t*) and *r*_2_ = *y*(*t*) to embed the chaotic operator into the canonical PSO method.

There are some other chaotic PSO methods proposed in the past. Wang *et al.* [29] proposed a chaotic PSO to find the high precision prediction for the grey forecasting model. Chuang *et al.* [30] proposed a chaotic catfish PSO for solving global numeric optimization problem. Araujo *et al.* [31] intertwined PSO with Lozi map chaotic sequences to obtain Takagi-Sugeno fuzzy model for representing dynamic behaviors. Coelho [32] presented an efficient PSO algorithm based on Gaussian distribution and chaotic sequence to solve the reliability–redundancy optimization problems. Coelho *et al.* [33] presented a quantum-inspired version of the PSO using the harmonic oscillator well to solve the economic dispatch problem. Cai *et al.* [34] developed a multi-objective chaotic PSO method to solve the environmental economic dispatch problems considering both economic and environmental issues. Coelho *et al.* [35] proposed three differential evolution approaches based on chaotic sequences using logistic equation for image enhancement process. Sun *et al.* [36] proposed a drift PSO and applied it in estimating the unknown parameters of chaotic dynamic system.

The main difference between our ACPSO and popular PSO lies in two points: (1) we introduced in the adaptive inertia weight factor strategy; (2) we used the Rossler attractor because of the following advantages [37]: the Rossler is simpler, having only one manifold, and easier to analyze qualitatively. In total, the procedures of ACPSO are listed as follows:

Step 1 Initialize every particle’s position with a uniformly distributed random vector;
Step 2 Initialize every particle’s best known position to its initial position, *viz.*, *p_i_* = *x_i_*;
Step 3 If *f*(*p_i_*) < *f*(*g*), then update the swarm’s best known position, *g* = *p_i_*;
Step 4 Repeat until certain termination criteria was met:
  Step 4.1 Update the value of ω according to formula (18);
  Step 4.2 Pick chaotic random numbers *r*_1_ & *r*_2_ according to formula (19)
  Step 4.3 Update every particle’s velocity according to formula (16);
  Step 4.4 Update every particle’s position according to formula (17);
  Step 4.5 If *f*(*x_i_*) < *f*(*p_i_*), then update the particle’s best known position, *p_i_* = *x_i_*. If *f*(*p_i_*) < *f*(*g*), then update the swarm’s best known position, *g* = *p_i_*.
Step 5 Output *g* which holds the best found solution.

### 4.3. ACPSO-NN

Let *ω*_1_, *ω*_2_, *ω*_3_ represent the connection weight matrix between the input layer and the first hidden layer, between the first and the second hidden layer, and between the second hidden layer and the output layer, respectively. When the ACPSO is employed to train the multi-layer neural network, each particle is denoted by:

(20) $$\omega \;=\;[\omega_{1},\;\omega_{2},\;\omega_{3}]$$

The outputs of all neurons in the first hidden layer are calculated by following steps:

(21) $$y_{1j}\;=\;f_{H}\;\left(\sum_{i=1}^{N_{I}}\omega_{1}\;(i,\;j)x_{i}\right)\;j\;=\;1,2,\;\cdots ,\;N_{H1}$$

Here *x_i_* denotes the *i*th input value, *y*_1_*_j_* denotes the *j*th output of the first hidden layer, and *f_H_* is referred to as the activation function of hidden layer. The outputs of all neurons in the second hidden layer are calculated as:

(22) $$y_{2k}\;=\;f_{H}\;\left(\sum_{j=1}^{N_{H1}}\omega_{2}\;(j,\;k)y_{1j}\right)\;k\;=\;1,2,\;\cdots ,\;N_{H2}$$

where *y*_2_*_j_* denotes the *j*th output of the second hidden layer.

The outputs of all neurons in the output layer are given as follows:

(23) $$O_{l}\;=\;f_{O}\;\left(\sum_{k=1}^{N_{H2}}\omega_{3}\;(k,\;l)y_{2k}\right)\;l\;=\;1,\;2,\ldots ,\;N_{O}$$

Here *f_O_* denotes the activation function of output layer, usually a line function. All weights are assigned with random values initially, and are modified by the delta rule according to the learning samples traditionally.

The error of one sample is expressed as the MSE of the difference between its output and the corresponding target value:

(24) $$E_{m}\;=\;\text{mse}\left(\sum_{l=1}^{N_{O}}\left(O_{l}\;-\;T_{l}\right)\right)\;m\;=\;1,\;2,\ldots N_{S}$$

where *T_k_* represents the *k*th value of the authentic values which are already known to users, and *N_S_* represents the number of samples. Suppose there are *N_S_* samples, then the fitness value is written as:

(25) $$F\;(\omega )\;=\;\sum_{m=1}^{N_{S}}E_{m}$$

where *ω* represents the vectorization of the (*ω*_1_, *ω*_2_, *ω*_3_). Our goal is to minimize this fitness function *F*(*ω*) by the proposed ACPSO method, viz., force the output values of each sample approximate to corresponding target values.

### 4.4. Cross Validation

**Figure 6.**
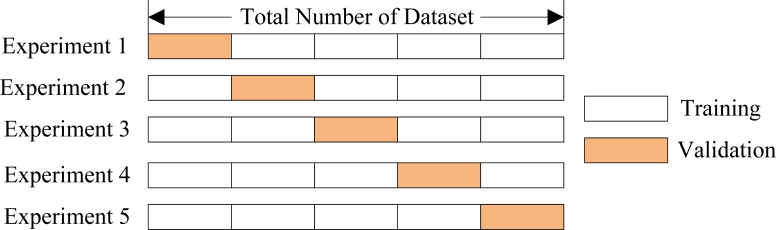
A 5-fold cross validation.

Cross validation methods consist of three types: Random subsampling, *K*-fold cross validation, and leave-one-out validation. The *K*-fold cross validation is applied due to its properties as simple, easy, and using all data for training and validation. The mechanism is to create a *K*-fold partition of the whole dataset, repeat *K* times to use *K*-1 folds for training and a left fold for validation, and finally average the error rates of *K* experiments. The schematic diagram of 5-fold cross validation is shown in Figure 6.

A challenge is to determine the number of folds. If *K* is set too large, the bias of the true error rate estimator will be small, however, the variance of the estimator will be large and the computation will be time-consuming. Alternatively, if *K* is set too small, the computation time will decrease, the variance of the estimator will be small, but the bias of the estimator will be large. The advantages and disadvantages of setting *K* large or small are listed in Table 3. In this study, *K* is determined as 10 through trial-and-error method.

If the model selection and true error estimation are computed simultaneously, the data needs to be divided into three disjoint sets [38]. In another word, the validation subset is used to tune the parameters of the neural network model, so another test subset is needed only to assess the performance of a trained neural network, *viz.*, the whole dataset is divided into three subsets with different purposes listed in Table 4. The reason why the validation set and testing set cannot merge with each other lies in that the error rate estimation via the validation data will be biased (smaller than the true error rate) since the validation set is used to tune the model [39].

## 5. Experiments

Flevoland, an agricultural area in The Netherlands, is chosen as the example. The site is composed of strips of rectangular agricultural fields. The scene is designated as a supersite for the earth observing system (EOS) program, and is continuously surveyed by the authorities.

### 5.1. Refine Lee Filter

**Figure 7.**
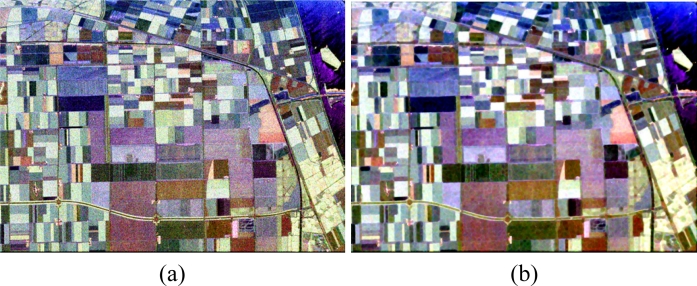
Pauli Image of Flevoland (1,024 × 750). (**a**) Pauli Image; (**b**) The refine Lee filtered images.

The Pauli image of Flevoland is shown in Figure 7(a), and the refine Lee filtered image (Window Size = 7) is shown in Figure 7(b).

### 5.2. Full Features

**Figure 8.**
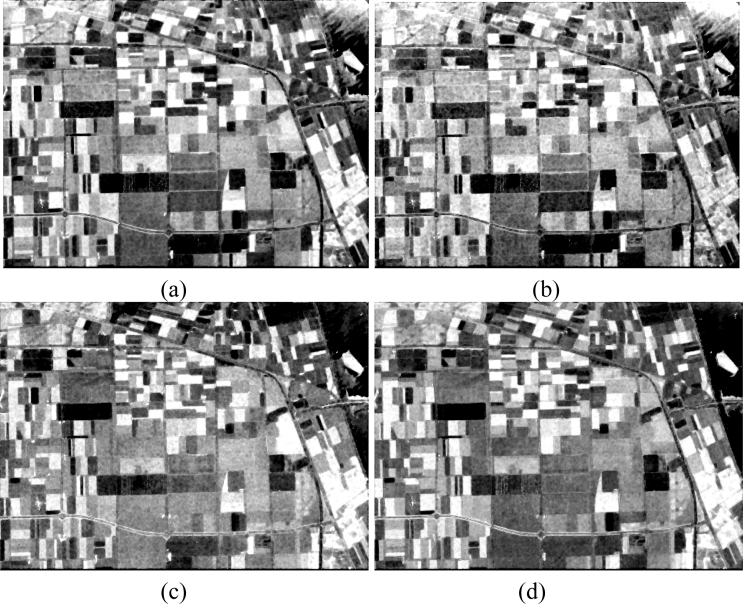
Basic span image and three channels image. (**a**) Span (dB); (**b**) *T*_11_ (dB); (**c**) *T*_22_ (dB); (**d**) *T*_33_(dB).

**Figure 9.**
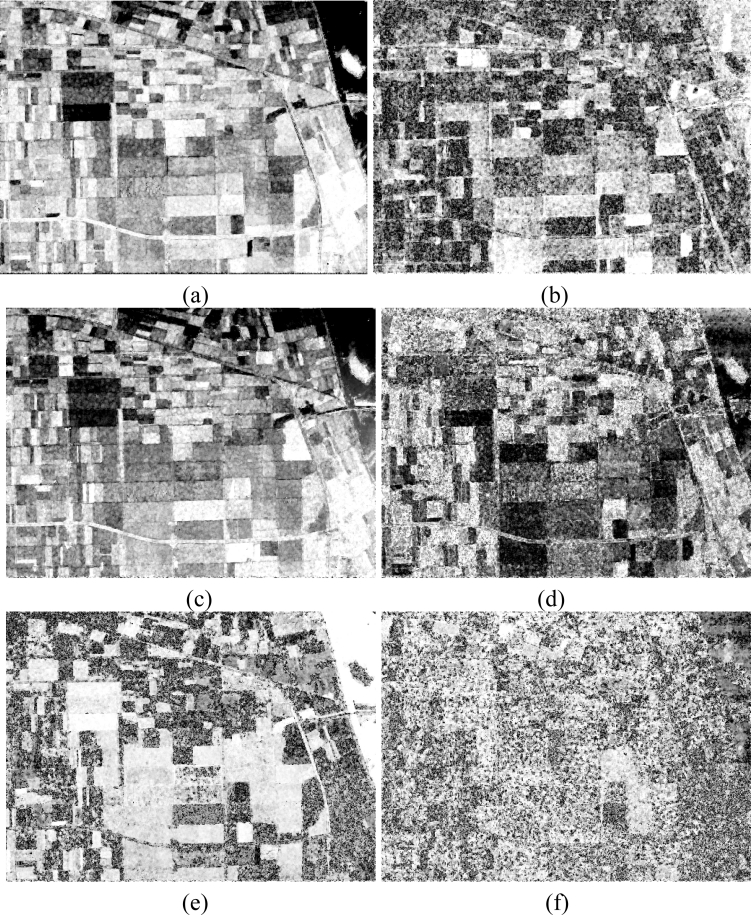
Parameters of H/A/α decomposition. (**a**) *H*; (**b**) *A*; (**c**) *ᾱ*; (**d**) *β̄*; (**e**) *δ̄*; (**f**) *γ̄*.

**Figure 10.**
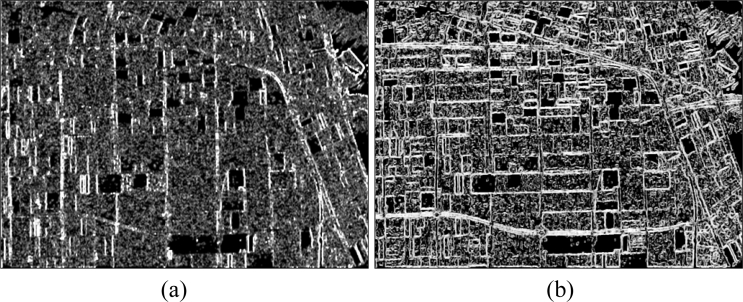

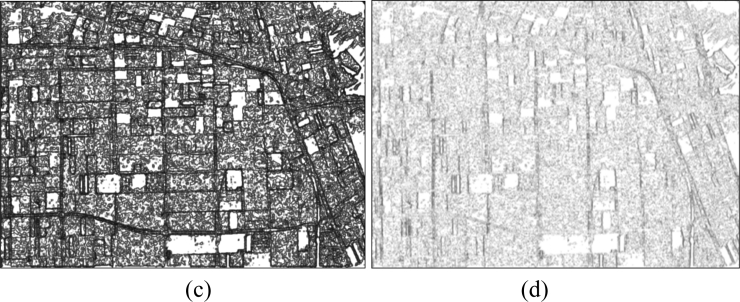
GLCM-based features of *T*_11_. (**a**) Contrast. (**b**) Correlation. (**c**) Energy. (**d**) Homogeneity.

**Figure 11.**
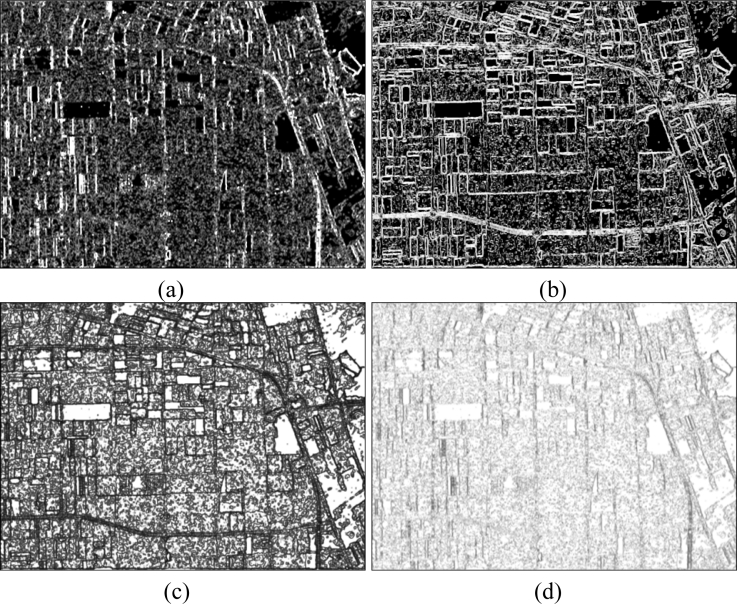
GLCM-based features of *T*_22_. (**a**) Contrast; (**b**) Correlation; (**c**) Energy; (**d**) Homogeneity.

**Figure 12.**
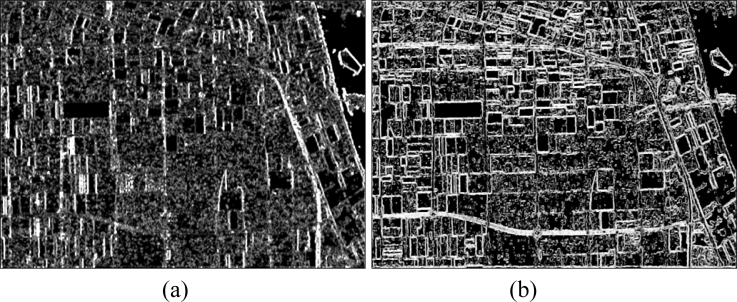

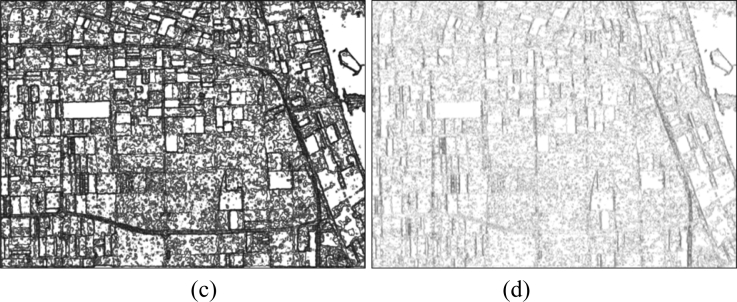
GLCM-based features of *T*_33_. (**a**) Contrast; (**b**) Correlation; (**c**) Energy; (**d**) Homogeneity.

The basic span image and three channels (*T*_11_, *T*_22_, *T*_33_) are easily obtained and shown in Figure 8. The parameters of H/A/Alpha decomposition are shown in Figure 9. The GLCM-based parameters of *T*_11_, *T*_22_, *T*_33_ are shown in Figures 10–12.

### 5.3. PCA

**Figure 13.**
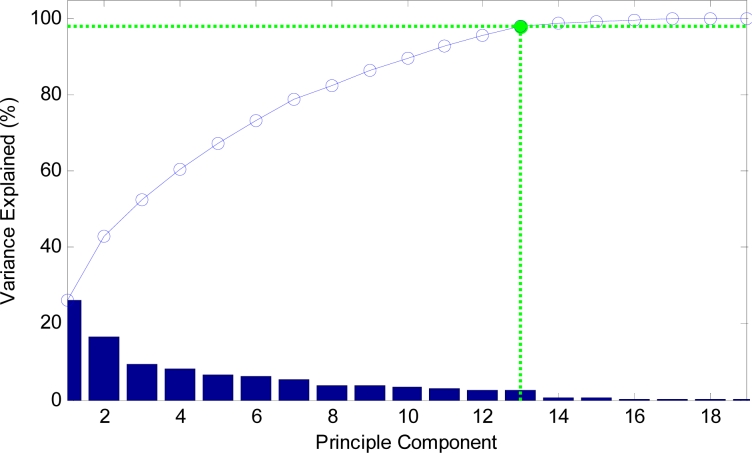
Cumulative sum of variance *versus* principle components.

The curve of cumulative sum of variance with dimensions of reduced vectors via PCA is shown in Figure 13. The detailed data are listed in Table 5. It shows that only 13 features, which are only half the original features, could preserve 98.06% of variance.

### 5.4. Area Selection

The classification is run over 13 classes, bare soil 1, bare soil 2, barley, forest, grass, lucerne, peas, potatoes, rapeseed, stem beans, sugar beet, water, and wheat. Our strategy is a semiautomatic method, viz. the training area was chosen and labeled manually. For each crop type, we choose a square of size 20 × 20, which is easy to perform since the training area size is 13 × 20 × 20 = 5,200 compared to the size of the whole image is 1,024 × 750 = 768,000. In order to reduce the complexity of experiment, the test areas are chosen randomly from rest areas [40,41], with the same square size as the training area.

**Figure 14.**
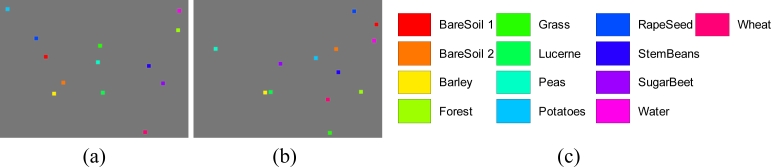
Sample data areas of Flevoland. (**a**) Training Area; (**b**) Test Area; (**c**) Legend of Colors.

The final manually selected training areas are shown in Figure 14(a). Each square corresponds to a crop type with the size of 20 × 20. In total, there are 5,200 pixels for our training. The cross validation procedures loop 10 times, therefore, each loop we use 4,680 pixels for training and the left 520 pixels for validation. The final randomly selected test areas are shown Figure 14(b). The samples numbers of training and test area are shown in Table 6.

### 5.5. Network Training

*N_I_* is determined as 13 due to the 13 features obtained by PCA. *No* is determined as 13 due to the 13 classes shown in Figure 14. Both *N_H_*_1_ and *N_H_*_2_ are set as 10 via the information entropy method [42]. Therefore, the number of unknown variables of the neural network is 13 × 10 + 10 + 10 × 10 + 10 + 10 × 13 + 13 = 393.

The network was trained by the proposed ACPSO algorithm, of which the parameters are obtained via trial-and-error method and shown in Table 7. Besides, BP algorithm [8], BP with momentum (MBP) [43], adaptive BP algorithm (ABP) [44], and PSO [45] are employed as comparative algorithms.

**Figure 15.**
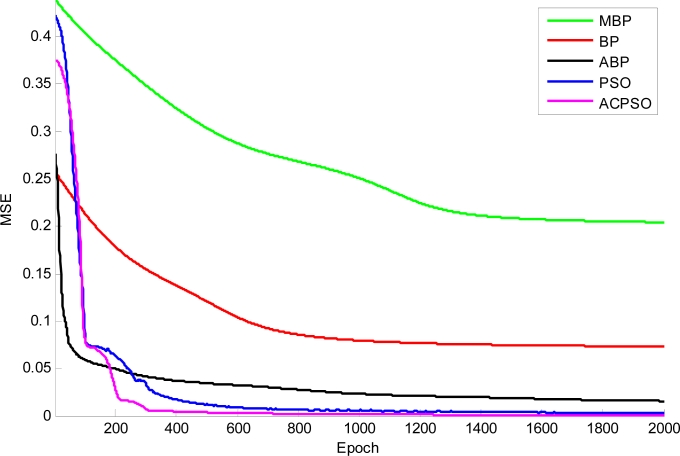
The curve of fitness *versus* epoch.

The curves of function fitness *versus versus* epoch of different algorithms are shown in Figure 15, indicating that the proposed ACPSO converges the most quickly and is capable of finding global minimum point.

### 5.6. Classification Accuracy

**Figure 16.**
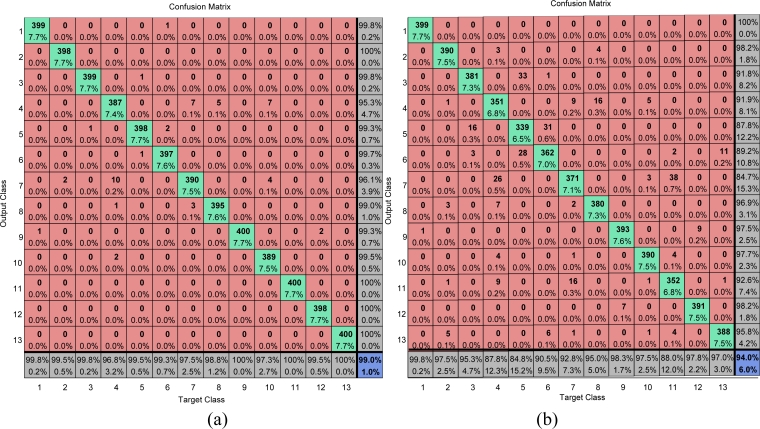
Confusion Matrixes of ACPSO-NN algorithm. (**a**) Training Area; (**b**) Test Area.

The confusion matrices on training area of our method are calculated and shown in Figure 16. The overall accuracies of our method on the training area (combining training and validation subsets) and test area are 99.0% and 94.0%, respectively. The main drawbacks are around the following four misclassifications: (I) forest zones are easily misclassified as peas; (II) grasses are easily misclassified as barley and lucerne; (III) lucerne are easily misclassified as grasses; (IV) sugarbeets are easily misclassified as peas.

A typical classification accuracy of both training area and test area by BP, ABP, MBP, and PSO are listed in Table 8, indicating that the proposed algorithm achieves the highest classification accuracy on both training (99.0%) and test area (94.0%). The random classifier disregards the information of the training data and returns random predictions, so it is usually employed to find the lowest classification rate.

Yudong also used Resilient back-propagation (RPROP) algorithm to train the neural network to classify the same Flevoland area [41], and obtains 98.62% on training area and 92.87% on test area. The PSO ranks the third with 98.1% on training area and 88.7% on test area. The ABP ranks the fourth with 90.7% and 86.4% on both training and test area, respectively. The BP and MBP performs the worst with the classification accuracy only a bit higher than the random classifier of 1/131 = 37. 69%, indicating that only 2,000 iterations are not enough for these two training strategies. Besides, the classification accuracy of the proposed algorithm was extremely high on the test area due to the 10-fold cross validation.

### 5.7. Robustness

In order to compare the robustness of each algorithm, we perform each algorithm 50 runs and calculated the minimum, the average, and the maximum of the classification rates. The results are listed in Table 9. It indicates that the results of each algorithm changed at each run, but the variation is limited, so the rank of the performance of all algorithms is the same as that in Table 8.

### 5.8. Time Analysis

Computation time is another important factor used to evaluate the classifier. The time for network training of our algorithm costs about 120 s, which can be ignored since the weights/biases of the NN remain fixed after training unless the property of images changes greatly. For example, the main crops in Flevoland are involved in the 13 types shown in Figure 14(c), therefore, the classifier can be directly used to other remote-sensing images of Flevoland without retrain. It will cost about 0.131 + 30, 0.242 + 40, 0.232 + 30, 0.181 + 80, 0.048 = 0.83 s from the input of Flevoland images (size 1,024 × 750) to the output of final classification results as shown in Table 10. For each pixel, it costs only 1.08 × 10^−7^s, which is fast enough for real time applications.

## 6. Conclusions

In this study, a crop classification classifier was constructed by following stages. First, a hybrid feature set was introduced which was made up of the span image, the H/A/α decomposition, and the GLCM-based texture features. Afterwards, PCA was carried on to reduce the features. The principle components were sent to the two-hidden-layer neural network, which was trained by the proposed ACPSO method. 10-fold cross validation was employed to prevent overfitting. Experiments on Flevoland site show that the proposed ACPSO-NN obtains satisfying results. The ACPSO trains the neural network more efficiently and effectively than BP, ABP, MBP, PSO, and RPROP methods. More rigorous testing on more complex problems will be performed in future works.

## Figures and Tables

**Table 1.** Pauli bases and their corresponding meanings.

**Pauli Basis**	**Meaning**
*S_a_*	Single- or odd-bounce scattering
*S_b_*	Double- or even-bounce scattering
*S_c_*	Those scatterers which are able to return the orthogonal polarization to the one of the incident wave (forest canopy)

**Table 2.** Properties of GLCM.

**Property**	**Description**	**Formula**
Contrast	Intensity contrast between a pixel and its neighbor	Σ|*i*–*j*|^2^*p*(*i*,*j*)
Correlation	Correlation between a pixel and its neighbor (μ denotes the expected value, and *σ* the standard variance)	Σ [(*i–μ_i_*)(*j–μ_j_*)*p*(*i*,*j*)/(*σ_i_σ_j_*)]
Energy	Energy of the whole image	Σ*p*^2^(*i*,*j*)
Homogeneity	Closeness of the distribution of GLCM to the diagonal	Σ[*p*(*i*,*j*)/(1+|*i*–*j*|]

**Table 3.** Large *K versus* small *K.*

***K* value**	**Estimator Bias**	**Estimator Variance**	**Computation Time**
Large	↓	↑	↑
small	↑	↓	↓

**Table 4.** Purposes of different subsets.

**Subset**	**Intent**
Training	Learning to fit the parameters of the classifier
Validation	Estimate the error rate to tune the parameters of the classifier
Testing	Estimate the true error rate to assess the classifier

**Table 5.** Detailed cumulative sum of variance.

**Dimensions**	1	2	3	4	5	6	7	8	9
**Variance (%)**	26.31	42.98	52.38	60.50	67.28	73.27	78.74	82.61	86.25

**Dimensions**	10	11	12	13	14	15	16	17	18
**Variance (%)**	89.52	92.72	95.50	98.06	98.79	99.24	99.63	99.94	99.97

**Table 6.** Sample numbers of training and test area.

**Training Area**	**Test Area**	**Total**
5,200 10 loops (4,680 for train and 520 for validation)	5,200	10,400

**Table 7.** Parameters of PSO & ACPSO.

**Parameters**	**Values**	
	**PSO**	**ACPSO**
Dimensions	393	393
*V*_max_	0.04	0.04
Maximum Iterations	2,000	2,000
*k*_max_	1,500	1,500
*NP*	24	24
*c*_1_	2	2
*c*_2_	2	2
Function tolerance	1e^−6^	1e^−6^
ω_max_	-	0.9
ω_min_	-	0.4
*a*	-	0.2
*b*	-	0.4
*c*	-	5.7

**Table 8.** A typical classification accuracy of different algorithms (Maximum iterations = 2,000).

**Algorithm**	**Training Area**	**Test Area**	**Rank**
Random	7.69%	7.69%	7
MBP	8.8%	7.5%	6
BP	8.3%	8.2%	5
ABP	90.7%	86.4%	4
PSO	98.1%	88.7%	3
RPROP[41]	98.62%	92.87%	2
ACPSO	99.0%	94.0	1

**Table 9.** Statistical results of different algorithms (Maximum iterations = 2,000).

**Algorithm**	**Training Area**			**Test Area**		

	**Min**	**Ave**	**Max**	**Min**	**Ave**	**Max**
Random	7.58%	7.69%	7.83%	7.58%	7.69%	7.81%
MBP	8.52%	8.83%	9.08%	6.98%	7.44%	7.92%
BP	7.96%	8.33%	8.65%	7.90%	8.17%	8.35%
ABP	81.04%	87.18%	94.12%	76.60%	83.55%	89.83%
PSO	95.83%	97.68%	98.52%	83.15%	89.32%	91.54%
RPROP	97.63%	98.71%	98.90%	90.87%	92.65%	93.77%
ACPSO	98.15%	98.84%	99.13%	92.56%	93.80%	94.52%

**Table 10.** Computation Time of Flevoland image classification.

**Stage**	**Time**
Span	0.13 s
H/A/α decomposition	0.24 s
GLCM	0.23 s
PCA	0.18 s
NN Training*	120 s
Classification	0.048 s

(* denotes training time can be ignored)
